# Enhanced flight performance by genetic manipulation of wing shape in *Drosophila*

**DOI:** 10.1038/ncomms10851

**Published:** 2016-03-01

**Authors:** Robert P. Ray, Toshiyuki Nakata, Per Henningsson, Richard J. Bomphrey

**Affiliations:** 1 School of Life Sciences, John Maynard Smith Building, University of Sussex, Falmer, Brighton BN1 9QG, UK; 2 Structure and Motion Laboratory, Royal Veterinary College, University of London, Hatfield AL9 7TA, UK; 3 Department of Biology, Lund University, Ecology Building SE-22362 Lund, Sweden

## Abstract

Insect wing shapes are remarkably diverse and the combination of shape and kinematics determines both aerial capabilities and power requirements. However, the contribution of any specific morphological feature to performance is not known. Using targeted RNA interference to modify wing shape far beyond the natural variation found within the population of a single species, we show a direct effect on flight performance that can be explained by physical modelling of the novel wing geometry. Our data show that altering the expression of a single gene can significantly enhance aerial agility and that the *Drosophila* wing shape is not, therefore, optimized for certain flight performance characteristics that are known to be important. Our technique points in a new direction for experiments on the evolution of performance specialities in animals.

## Abstract (web summary)

Insect wings are under multiple competing selection pressures, but which are important in natural populations is not clear. Using RNAi to modify wing shape, Ray *et al.* show that aerial agility can be significantly enhanced in *Drosophila*, suggesting that natural variation does not reflect an optimization solely for flight agility.

## Graphical abstract

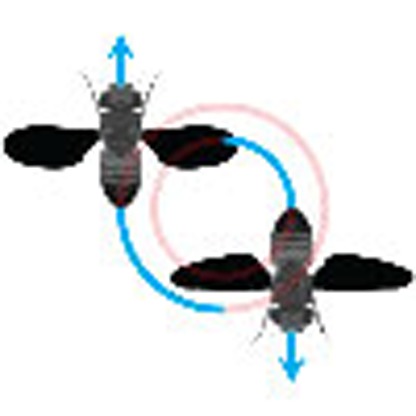

Animal morphologies reflect the cumulative effect of non-adaptive variation and time-integrated selective pressures including – but not limited to – those optimizing form for function^1^. Insect wings are under selective pressures driving towards local multi-objective optima, embodying a design compromise between features that are aerodynamically relevant (contributing to flight performance) and features that may contribute to fitness but are independent of aerobatic capability. They are also shaped by non-adaptive influences and may exhibit some features that are unrelated to fitness. Insects do not have the same capacity as birds and bats to alter wing shape^2^ and affect the resultant aerodynamics^3^ during flight and, as such, they represent an attractive model system with which to investigate directly the effect of functional morphology on aspects of aerodynamics^4^,^5^,^6^,^7^,^8^,^9^, power^10^,^11^,^12^ and performance^6^,^13^,^14^,^15^.

Until now, studies of comparative flight performance in insects have involved trimming the wings of a model species^12^, intensive selection over multiple generations^16^, or sampling across many species^17^, each of which introduces confounding factors. Cutting the wings reduces their area, removes sensilla that may contribute to flight stability and control, and exposes the wing veins to desiccation. Selective breeding can be successful in altering either performance or morphology but simultaneously changes multiple genotypic and phenotypic factors. In so doing, the mechanistic basis for the observed behavioural change remains hidden and difficult to characterize. A powerful comparative analysis is time consuming to execute, requires detailed knowledge of the phylogeny and is complicated further by the standardization of experiments where environment-dependent flight performance is likely to be species-specific (for example, preferred light intensities or temperatures).

In this study we take a different approach and use genetic manipulation to modify wing shape in the fruit fly *Drosophila melanogaster*. Using the Gal4/UAS system and RNA interference (RNAi), we knockdown expression of a single gene which leads to dramatic shape changes that are expected, from first principles, to have important consequences for flight. By restricting the RNAi to the developing wing, we isolate the effects of wing planform from other confounding variables and can directly address how wing shape affects flight performance. Our results show that large and small changes in shape can have significant effects on key performance metrics, sometimes resulting in improved manoeuvrability and agility. Physical modelling predicts that this increased performance comes at a cost that ultimately supersedes the benefit, rendering the performance of the most extreme wing shapes equal to or less than that of the wild-type wing shape. The development of this technique opens the door for further studies on the effects of wing shape and structure on flight performance.

## Results

### Wing shape change

**Figure 1:**
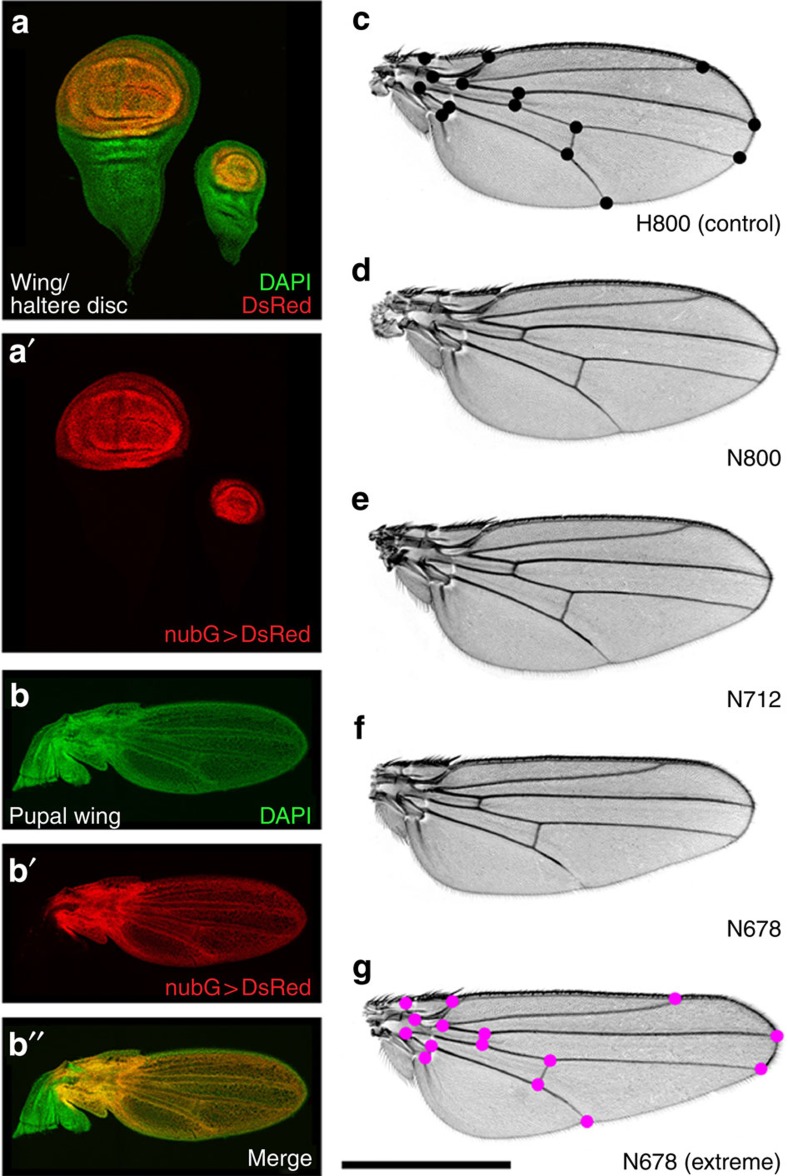
Changing wing shape with *nub-Gal4* and *nw-RNAi*. (**a**) Larval wing (left) and haltere (right) discs showing the expression pattern of the *nub-Gal4* with the reporter *UAS-His2ADsRed*. Images are a full projection of the disc showing all nuclei in green and the *nub-Gal4>UAS-His2ADsRed* in red. (**b**) Expression of *nub-Gal4* at 28 h APF when the wing has reached the ‘definitive stage' where it resembles the adult wing in every respect but size. *nub-Gal4* expression includes all wing blade cells, but stops at the junction with the hinge. (**c**–**g**) Adult wings from CONT (**c**), N800 (**d**), N712 (**e**) and N678 (**f**) genotypes, each image representing the wing closest to the mean PC1 coefficient for the genotype. (**g**) shows the wing with the most extreme PC1 coefficient from the N678 genotype. (**c**,**g**) highlight the fifteen landmarks used in our principal component analysis. With increasing morphometric severity, landmarks at the tip of the wing move distally, while anterior and posterior landmarks move closer to the medial axis resulting in a more tapered wing with higher aspect ratio. Scale bar, 1 mm (**c**–**g**).

Over the last century, a host of *Drosophila* genes have been identified that affect wing shape^18^,^19^. While many of these affect wing patterning as well as shape, a small class, including the genes *narrow*, *tapered*, and *lanceolate*, affect wing shape without affecting pattern^19^ (Fig. 1). We reasoned that if these genes could be inactivated exclusively in the developing wing blade, we could use the resulting flies to test non-invasively the effect of wing shape on flight performance. To achieve this, we have used the Gal4-UAS system to drive three distinct RNAi constructs against the *narrow* (*nw*) gene in the developing wing. To restrict the RNAi to the developing wing, we used *nubbin-Gal4* (*nub-Gal4*), which drives expression at high levels in the developing wing and haltere discs, but at negligible levels in other tissues^20^ (Fig. 1; Supplementary Fig. 1).

In the wing disc, *nub-Gal4* expression is restricted to the wing pouch, corresponding to the wing blade proper, and not in the surrounding notum tissue that forms the wing hinge and thorax (Fig. 1a,b). RNAi of *nw* using the *nub-Gal4* driver gives rise to a characteristic wing shape defect but has no effect on the structure of the hinge or notum, and, importantly, no effect on the shape of the haltere (Supplementary Fig. 2), presumably reflecting a different requirement for *nw* in these two tissues. For our experiments, three different *nw-RNAi* hairpin constructs were used to produce a nearly continuous range of wing morphs (Fig. 1), and four different genotypes were tested, which we refer to as CONT (control), N800, N712 and N678, that together sample the entire phenotypic range from wild type (CONT), to the two milder phenotypes (N800 and N712), and the most severe (N678) shape changes.

**Figure 2:**
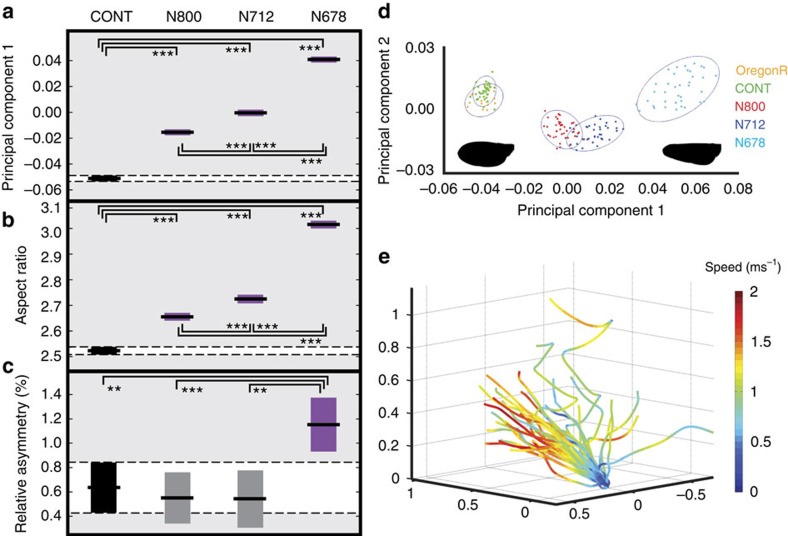
Morphological change across the experimental groups with increasing phenotypic strength. Boxes show 95% confidence intervals with median values and are coloured purple if the metric was significantly different from CONT. (**a**) single wing coefficients for Principal Component 1, (**b**) single wing aspect ratio (defined as length^2^ per area), (**c**) relative asymmetry (defined as the percentage increase of the longer wing over the shorter wing. Flies are grouped by genotype with significant pairwise comparisons coloured based on Tukey's honestly significant difference criterion, which puts an upper bound on the probability that any comparison will be incorrectly found significant. *Post hoc* pairwise ANOVA shows the degree of significance for groups found to be different under the Tukey criterion (*n*=85; *P≤0.05; **P≤0.01; ***P<0.001). (**d**) Geometric morphometric analysis of all wing shapes for which a full set of landmarks could be collected (including both left and right). Analysis using fifteen landmarks (Fig. 1) shows that principal component 1 (PC1) is monotonic with the severity of the phenotype and explains 83.0% of the variation in wing shape. PC2 explains 7.8%. Control flies (CONT) overlie the Oregon R background line. Silhouettes show wing outlines from the most distant phenotypes along the PC1 axis used in flight tests. (**e**) The first two recorded flight trajectories from each of the 21 flies in the control group as they explore the arena, coloured by flight speed.

The different *nub-Gal4>nw-RNAi* combinations resulted in a spectrum of wing morphs with a higher aspect ratio than wild type (Fig. 2a,b). A principal component analysis using wing vein junction landmarks^21^ revealed that 83% of the phenotypic variance is described by the first principal component (PC1; Fig. 2d), 7.8% by the second and 2.8% by the third (Supplementary Fig. 3). Of these, only PC1 is both substantially modified (analysis of variance (ANOVA); *n*=85, *F*=922.6, *P*<0.001) and monotonic with phenotypic severity. This statistical model, and those that follow, applies an ANOVA to the scores for each fly using genotype as a fixed factor. PC1 is a warp that separated the genotypes and can be thought of in simplified terms as an increase in the wing aspect ratio by tapering and reducing the mean chord length (Fig. 1, Fig. 2a,b and Table 1).

### Flight performance

Having identified substantial differences in wing morphology, we flew the four groups of flies in a standardized arena. As we sought to elicit typical rather than maximal performance, the maxima and minima refer to the 99th percentile values observed during routine exploratory flights in our flight arena. Soliciting truly maximal performance from animals is challenging and risks introducing unknown variables into the analysis pertaining to the individual's degree of motivation to complete a task. Attempts to determine performance assays have been necessarily limited in either their certainty that maximal performance has indeed been achieved, or the scope of the behavioural repertoire that is measured. The three principal methods that have been used for flies are: presentation of optomotor^22^ or mechanical^23^ tasks to flies that are motivated to light or food; presentation of an artificial looming stimulus^15^ or alternative collision avoidance cue, for example a patterned wall^24^,^25^; or the introduction of a predator^26^. Unidirectional tasks such as wind tunnel assays^16^,^23^, or single axis translational optomotor tasks^22^ are poor assays for turning performance, and looming response assays naturally focus on evasive turns. Even in the case of well-described, reliable responses to looming stimuli, there remains large variation in gain, measured, for instance, by the change in heading angle when compared with the stimulus angle^27^.

The most biologically relevant of the three options is the introduction of a predator in a laboratory-based enactment of a crucial juncture in the life of a fruit fly. In this scenario, the fly's strategy is reported to be a reliance on its routine turning behaviour^26^. For these reasons, we believe our experimental paradigm is a reasonable approximation of fly performance during exploration and foraging, and is sufficiently well-sampled so as to eliminate variability in the motivation of individuals from being a confounding factor. The extent of our sampling can be seen in Supplementary Fig. 4, with more than half a million camera images yielding 264,000 positional points. Previous experiments have recorded for longer durations^24^,^25^,^27^ but at substantially lower temporal resolutions that cannot provide the positional information we required to calculate turn radii.

When tracking the flies in a large arena (Fig. 2e), the control group demonstrated flight performance comparable with existing measurements^26^ (Table 1). The flies travelled at a mean speed of 0.71±0.33 ms^−1^ but were capable of speeds up to 1.60±0.16 ms^−1^. They accelerated tangentially at 6.60±1.23 ms^−2^ and decelerated at 7.68±2.83 ms^−2^. When turning, the flies were able to corner around a radius of just 13.2±5.9 mm, with a modal turn radius of 85.9±28.5 mm. The maximum turn rates we observed, based upon the change in three-dimensional (3D) trajectory angle, of 1,427±378 deg. s^−1^ were marginally higher than those reported elsewhere for free flying fruit flies^26^ and somewhat lower than the peak turn rates of flies responding to looming stimuli^15^.

**Figure 3:**
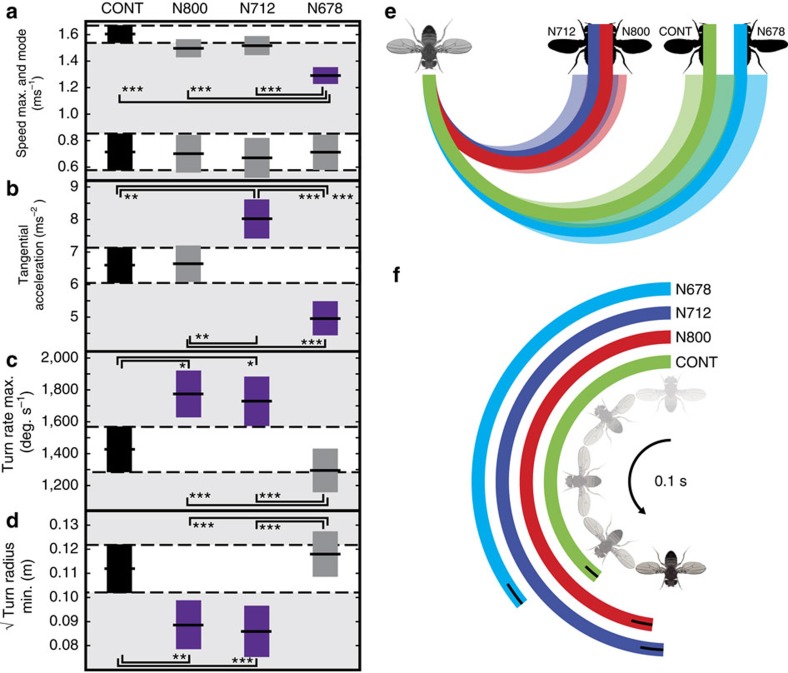
Modulation of flight performance. Boxes show 95% confidence intervals with median values and are coloured purple if the performance was enhanced or diminished relative to the control group. (**a**) peak and modal flight speeds, (**b**) peak tangential accelerations (**c**) maximum turn rate and (**d**) minimum observed turn radius. Flies are grouped by genotype with significant pairwise comparisons coloured based on Tukey's honestly significant difference criterion (*n*=85; *P≤0.05; **P≤0.01; ***P<0.001). (**e**) Differences in turn radii shown as if the flies from each group were to execute 180 degree turns with their minimum observed (99th centile) turn radius. Shading shows 95% confidence intervals. (**f**) Differences in heading if the flies were to turn at their maximum observed turn rate for 0.1 s. Black bars show the 95% confidence intervals.

Remarkably, we observed enhanced performance in several important agility metrics in both of the two milder genotypes (N800, N712) relative to the control group. While maximal and modal flight speed did not change, the N712 flies exhibited significantly improved tangential acceleration (N712, +21.5%) and deceleration (N800, +9.8%; N712, +27.0%). Moreover, when turning, angular rate increased (N800, +24.3%; N712, +21.1%) and turn radius decreased (N800, –0.8%; N712, –23.3%) indicating superior agility and manoeuvrability (Fig. 3; Supplementary Table 1). As expected, neither modal nor maximal centripetal accelerations varied between groups (except in the case of the most extreme morph's maximal centripetal acceleration; Supplementary Table 1) because the flight motor is unchanged. Here, centripetal acceleration is calculated as the square of the velocity divided by the turn radius. The relationship between turn rate, turn radius and centripetal acceleration is presented in Supplementary Fig. 5. Thus, it is the shape changes associated with the N800 and N712 genotypes that improves turning ability but not at the expense of straight line speed or acceleration.

**Figure 4:**
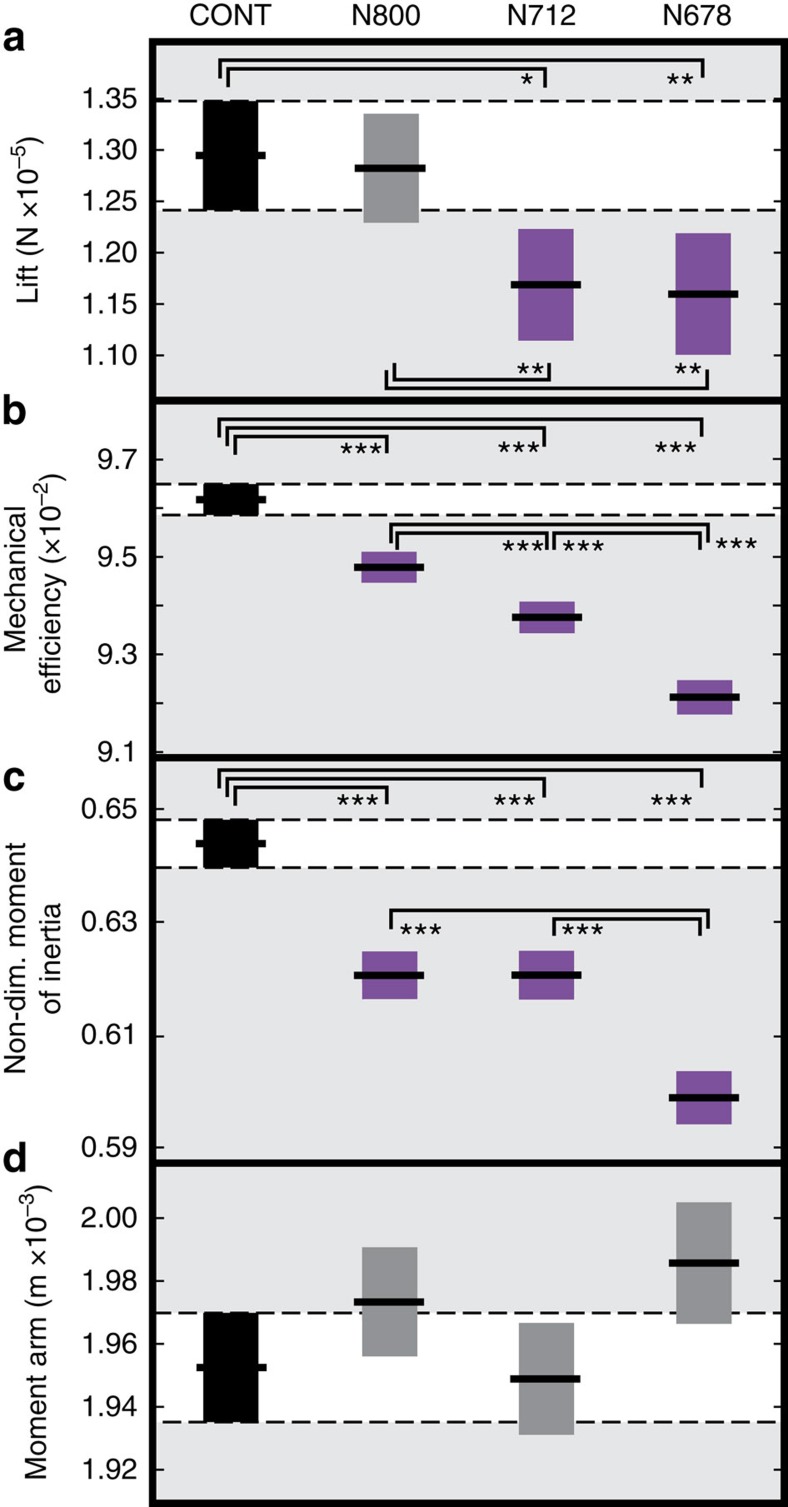
Calculated aerodynamic and mechanical variation between groups. (**a**) Estimate of lift based on a blade element model with standard wingbeat kinematics, (**b**) estimate of mechanical efficiency, *h*, using blade element analysis and Rankine–Froude momentum theory, (**c**) estimate of the wing non-dimensional moment of inertia around the wing hinge assuming constant thickness and density, (**d**) estimate of the wing centre of pressure moment arm using the blade element model. Boxes show 95% confidence intervals with median values and are coloured purple if significantly enhanced or diminished relative to the control group.

The improved turning performance we observed with the N800 and N712 genotypes is unexpected given that aircraft theory would predict that increasing aspect ratio should have an adverse effect on turning due to the effect of moving wing mass further away from the centre of mass. To address this, we used a combination of analytical aerodynamic theory and classical mechanics to predict the effect of our novel morphs on aerodynamic performance and agility (see Methods). For each fly, we calculated the length of the moment arm and the wing moment of inertia assuming uniform wing thickness and density^28^. Significantly, the structural model predicts that, while the moment arm remains constant across all genotypes, the non-dimensional moment of inertia decreases monotonically with phenotypic severity (Fig. 4c,d). Thus, so long as muscle power is not limiting, aerodynamic torque remains constant while the non-dimensional moment of inertia decreases. This results in greater angular accelerations and better turning performance. Notably, this effect is a result of the specific shape change that is produced by the *nw-RNAi*: if the shape warp were a simple narrowing of the wing, the mechanics of aircraft theory would prevail, but because the novel wings taper, the increase in aspect ratio is accompanied by a inboard shift in the wing centre of mass resulting in increased agility.

## Discussion

Insect flight performance is a direct consequence of the interaction of the wings with the air and is determined by a combination of kinematics and morphology. Routine behaviour can be dominant over escape responses as the predictor of survival in dragonfly-fruit fly interactions, with sharp turns highlighted as vital for evading capture^26^. Selective pressures on fruit fly morphology may have been expected, therefore, to promote adaptations that enable a high degree of manoeuvrability. Our findings show that fruit flies do not develop wings that are best suited for agile flight even when driven by their existing flight motor. Moreover, flight performance envelopes could be widened by affecting the function of a single gene. That flies are suboptimal in this regard is not particularly surprising but symptomatic of at least one antagonistic developmental, physical or behavioural selection pressure: for example, sexual selection mediated by the effect of wing planform on auditory or visual cues^29^.

Flight agility could not be improved indefinitely. In the most severe morph, if significant differences were observed, flight performance was always reduced in comparison with the N800 and N712 morphs and, in some cases, with the control (Fig. 3). Indeed, despite the fact that our non-dimensional moment of inertia estimate predicts improving turning performance across all the morphs (Fig. 4c), both turn rate and turn radius reverted to control levels in the most extreme morphs (N678; Fig. 3c,d; Supplementary Table 1). To explain this divergence from our prediction in N678, we estimated the aerodynamic mechanical efficiency for a single wing, measured from each individual used in the flight performance tests, using Rankine–Froude momentum theory and a blade element model based on standardized kinematics and coefficients of lift and drag^5^,^6^,^10^,^28^.

The aerodynamic model predicts that aerodynamic efficiency decreases monotonically with phenotypic severity, matching the patterns shown for PC1 and aspect ratio. The aerodynamic model also predicts that the lift force decreases between the N800 and N712 groups, which reflects the change in wing area and indicates that kinematic adjustment, and increased power, must be required to support the weight of the flies during flight (Fig. 4a,b). The wing area decreases between the N712 and N678 groups while the body mass remains constant. The classical aerodynamic prediction of increased wing loading would be an increase in flight speed but we do not observe this effect, indicating that wing loading is not a dominant effect. Changes in the kinematic pattern do not comprise adjusting wingbeat frequencies, which remain the same (Supplementary Table 1). Adjustments are therefore likely to involve either larger stroke amplitudes, or more rapid wing rotations. These, combined with an ultimate need for greater force generation, especially in the N712 and N800 morphs, are highly likely to increase the power required for flight still further and reduce efficiency more than that predicted by our standardized kinematics model. Thus, the improved flight performance may come at a cost: the shape changes that improve agility make it progressively more demanding to fly.

Our aerodynamic model shows that all of the wing morphs we have tested show a decrease in aerodynamic efficiency in comparison with the control. Thus, measurable differences in flight performance are likely to be the result of a balance between the aerodynamic and mechanical modifications due to the shape change and its energetic cost. In the two milder morphs, we suggest that the energetic cost is not high enough to negate the performance benefits whereas, in the most extreme morph, an intersection is crossed beyond which the necessary power is more challenging to achieve. A key factor contributing to this shift from improved to inferior performance is likely to be the disruption we have introduced into the system by altering the wing planform without changing the complex musculoskeletal apparatus that drives it^30^. In an engineering sense, the induced changes in wing shape force the flight motor to operate off-design. In the two milder wing morphs, the shape change is within the tolerance limits of the existing flight motor (though this may still be suboptimal) while in the extreme morph, the disparity between the motor and the wing planform may be too great and efficiency and functionality are diminished. A second factor that will also contribute to the poor performance observed in the most extreme morph flies is wing asymmetry. Asymmetry has predictable effects on flying animals, including changing the total span and shifting the centre of pressure laterally^31^. In the most severe phenotype there is an increase in asymmetry to 1.15% (N678, +81.3%; Fig. 2e; Supplementary Table 1) although this still represents a small difference in comparison with the large scale shape changes.

RNAi-mediated developmental control provides a platform to investigate systematically a large morphological parameter space, provided suitable genotypes can be manufactured. We did not encounter a physical limit along the principal component axis that we were able to influence; all our genotypes were able to fly, albeit with reduced performance maxima at the extremes of our morphological manipulations. We have shown that we can experimentally modify functional anatomy and measure the resulting modulation in locomotor performance. Using this method we were able to sidestep the rigid developmental control^18^,^32^ that would otherwise prevent sampling a range of morphologies within a Dipteran species. As such, we present a new model system with which to investigate the biomechanics of locomotion and the effect of evolutionary pressures acting on performance and morphology.

## Methods

### *Drosophila* strains

Flies were raised at 25 °C on standard *Drosophila* cornmeal-molasses medium. RNAi lines directed against the *CG43146* locus, corresponding to *narrow*, were obtained from the Vienna Drosophila RNAi Centre (VDRC). The three lines used in this study (v12800, v50712 and v49678, corresponding to the genotypes N800, N512 and N678, respectively) were driven with the Gal4/UAS system^33^, and expression was restricted to the developing wing with the *nubbin-Gal4* (*nub-Gal4*) driver^20^. The UAS-His2ADsRed reporter used to analyse the expression pattern of *nub-Gal4* was obtained from the Bloomington Stock Center.

Because the Gal4-UAS system is bipartite and requires a cross to unite the Gal4 and UAS constructs in a single fly, rather than performing the experiments in a pure isogenic background, we opted for a heterozygote between two isogenic strains. The RNAi lines were generated in an isogenic *w*^*1118*^ background^34^, and the *nub*-Gal4 insertion was introgressed into an isogenic Oregon R background. To generate the flies with different wing planforms, the *nub*-Gal4 strain was crossed to each of the three RNAi lines of the genotype *w*^*1118*^; UAS=CG*RNAi, and females of the genotype *w*^*1118*^*/+*; nub-Gal4/UAS=CG*RNAi, fully heterozygous for all chromosomes, were used in the experiment.

To control for the health and age of the flies, five independent crosses of five *w*^*1118*^; UAS=RNAi* males to eight *nub-Gal4* virgin females were made in vials and transferred daily to prevent crowding. Two days after the first flies had begun to eclose, the female progeny of the appropriate genotype were collected and placed into clean vials sprinkled with yeast. Flies were transferred daily into new vials until they were flight tested. All flights were made between 4 and 12 days after eclosion.

### Wing mounting

After completion of the flight experiments, flies were preserved in Isopropanol (Fisher). The wings were then dissected from the body and mounted in DPX mounting medium (Fisher) and photographed (Zeiss Axiophot: 2,592 × 1,944 pixels). Wing images were then analysed using two methods to describe variation in their shapes.

### Traditional morphometrics

We used custom written software (Matlab, Mathworks, MA, USA) to determine gross morphological variables using the wing margin. The start point was the anterior end of the humeral cross vein; the end point was identified as the junction between the alula and the posterior margin. Finally, the wing tip was identified as the terminus of radial vein L3. The wing outline was used to estimate wing area and aspect ratio. In this standardized procedure, the alula was not included in wing area estimates.

### Geometric morphometrics

Fifteen landmarks were digitized from wing images using the Fly Wing Kit plug-in for ImageJ provided by C. Klingenberg (Fig. 1) and geometric morphometric analyses were performed using MorphoJ^35^. Shape information for each genotype was extracted by Procrustes superimposition and outliers removed. Data for the different genotypes were then combined and a covariance matrix was generated that was used in a principal component analysis to quantify shape change.

### Trajectory data acquisition

We recorded self-motivated flights in a calibrated arena measuring 2 × 2 × 1.8 m (c.1,000 body lengths in each dimension) with a pair of synchronized, high-speed, CMOS cameras operating at 500 fps (Photron Fastcam SA3, Photron Europe Ltd, Bucks, UK). The flies were chilled on ice for ∼20 s then weighed (UMX2 ultra-microbalance; Mettler-Toledo, Leicester, UK) and allowed 30 min to recover. The arena was illuminated with DC lights (Arri CT limited, Middlesex, UK; Unomat International, Germany) and one netted wall provided camera access and visual cues for the flies in the form of equipment and furniture. Additional high contrast visual information came from a monochrome calibration grid (14 × 14 large dots; 59 mm spacing) laid on the floor.

The vials were opened 0.1 m above the grid; the flies then climbed to the rim and took off voluntarily. Flies tended towards the side of the arena with the greatest visual information so, to maximize the length of recorded sequences, take-off location was close to the back of the arena. Thus, flight trajectories tend to be largely upward and forward within the arena although we also captured downward flight and many turns. For each genotype, we aimed to record five flights from at least 20 flies of each phenotype. Flies that did not reach the top of the vial or who failed to take off after five minutes on two successive trials were discarded. In total we gathered c. 8.8 min of flight, which sampled the fly genotypes sufficiently for subsequent analysis (see Supplementary Fig. 4 for example histograms).

### Trajectory data processing

We used photogrammetry to reconstruct 3D trajectories as described elsewhere^14^,^36^. The following describes the salient features of the method and any modifications. The reader is referred to ref. 14 for further detail. First, we calibrated the volume with multiple images (c. 35) of a grid of known dimensions to minimize the reprojected pixel error using a bundle adjustment nonlinear least squares optimization routine^14^,^36^. The modal reprojected pixel error of the calibrations averaged 0.76 pixels. This resulted in a calibration matrix required for the next stage. The next step in the procedure used custom point tracking code (Matlab, Mathworks, MA, USA) to identify the two-dimensional co-ordinates of the fly in each camera view. These data were used in conjunction with the calibration matrix to reconstruct the 3D fly positions at each time interval. A quintic spline was fitted to the *x*, *y* and *z* co-ordinates so that differentiation would yield an analytical solution for velocity and acceleration. The smoothing tolerance was calculated from the residuals of a third-order Butterworth filter. The tolerance factor for *SPAPS* was 1.1 × the sum of the filter residuals squared. The initial Butterworth cut off frequency of 30 Hz was selected by an autocorrelation method described previously that evaluates autocorrelation in the residuals compared with autocorrelation of the residuals from white noise filtered in the same way^14^. The data were padded at the start and end points using reflection around the boundary point to reduce errors at the start and end of the data series. We tested the procedure by dropping an object of comparable dimensions to the fly in the centre and at four extremities of the calibrated volume and subsequently compared the calculated acceleration with standard gravity, *g*. The mean acceleration of the ball immediately after release was calculated to be 9.730 ±0.070 ms^−2^: an error of 0.78%.

### Wingbeat frequency acquisition

We filmed fly lines in a smaller chamber measuring 220 × 180 × 300 mm to assess wingbeat frequency. Flies performed self-motivated flights from a vial and were recorded using a single Phantom SA3 camera operating at 4,000 fps until they left the field of view. The first 10 wingbeats following take off were ignored and the remaining complete wingbeats counted, beginning and ending at dorsal stroke reversal (pronation). We recorded 10 flies per genotype, aiming to capture a minimum of 50 wingbeats per fly; the mean number was 114 ±38.6. Wingbeat frequency for each fly was then normalized by its mass for inter-genotype comparison. Flights were recorded in a constant temperature room at 29° (Supplementary Table 1).

### Geometric morphometrics using principal component analysis

We chose to describe the RNAi-induced wing shapes using traditional morphological measurements as well as landmark-based, geometric morphometrics^35^. The former yields data that are fundamental to well-established aerodynamic theory, such as span length, chord length and aspect ratio. For those aerodynamic analyses, the detail of the wing vein architecture that supports the planform is inconsequential. The latter yields the main features of shape variation described by orthogonal principal components, or shape warps. The landmark-based approach is indispensable if we are to reveal the developmental mechanism underpinning the resultant wing phenotype. Furthermore, noting the proximal direction of movement of the relatively heavy wing veins, and the junctions between them, supports the notion that our estimate of the decreasing moment of inertia is a conservative one. Wing shape is governed by the expression of a number of genes, the modulation of which leads to phenotypic variation. The results of changing the balance of expression bears little relation to the independent parameters engineers vary when designing aircraft wings, yet these are the gene expression-driven shape warps upon which natural selection acts.

### Calculating the second moment of wing area

We calculated the second moment of wing area for a single wing from each individual. The *n*th moment of wing area, *S*_*n*_, is defined by


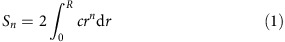


where *R* is wing length, *c* is local chord length and *r* is radial position along the wing^28^. If we assume uniform density and thickness, the moment of inertia of wings *I*_w_ is given by


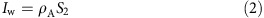


where *ρ*_A_ is the wing mass per unit wing area, estimated to be 1.31 μg mm^−2^ according to the wing area and mass measured by Bergou *et al.*^13^. The wing's contribution to the moment of inertia about the body axis (and to the flight dynamics) is noticeable in insects such as the dronefly, in which the moment of inertia of the wings is about 30% of that of the body^37^. As the body's moment of inertia around the roll axis in *Drosophila*^38^ is 1.1 × 10^−13^ N m s^2^, the wings' moment of inertia reaches up to 10% of that of the body.

Here, in order to isolate the shape effect, the *S*_2_, and hence *I*_w_, are normalized by wing area *S* and wing length as follows^28^:


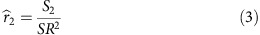


The 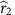 decreases when transitioning between the CONT and N800, and between N712 and N678, which can lead the enhancement of turning performance N800 and N712. It should be noted that, considering the shift of cross-veins towards the wing base and the distal tapering of veins, the estimated differences in the non-dimensional moment of inertia are a minimum bracket owing to our assumption of uniform density and constant thickness.

### Quasi-steady estimates of aerodynamic performance

The aerodynamic performance of a single wing from each individual during flapping flight is estimated using a blade element model under the quasi-steady assumption. A blade element model calculates aerodynamic force by integrating the forces on virtual chordwise blades. Translational force can be calculated by:


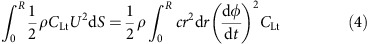


where *ρ*, *C*_Lt_, *U*, *φ*, and d*S* are density of air, the translational lift coefficient, velocity of the blade, wing's positional angle and the area of the blade, respectively; the aerodynamic force is proportional to the second moment of wing area^10^.

To isolate the effect of wing shape, all of the wings are evaluated with the same wing kinematics during hovering flight as measured by Fry *et al.*^39^. The time series of positional angle and angle of attack (feathering angle) are interpolated by 3rd order Fourier series. Wingbeat frequency *f* is set to 272 Hz. Mean vertical force is represented as two force components: translational force *F*_L,trans_, and rotational force *F*_L,rot_, which are calculated as follows:


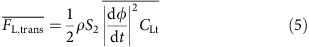



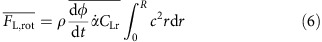


where *C*_Lt_ is given by Dickinson *et al.*^4^ as a function of angle of attack *α*. The single value 1.55 is used as the rotational force coefficient *C*_Lr_ (ref. 40). The effect of added mass is neglected here because its contribution to net force is quite small^41^. As expected from the changes in second moment of wing area, the aerodynamic force generation is significantly decreased when transitioning between the CONT and N800 strains and the N712 and N678 strains.

Aerodynamic efficiency is also estimated by the blade element analysis with Rankine–Froude momentum theory. Rankine–Froude estimates of induced power *P*_RF_, which is the minimum power required to generate the lift *F*_L_ (ref. 5), is given by:


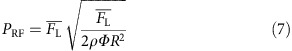


The induced power *P*_ind_, which depends on the induced velocities of the wake in association with lift, is given with a correction factor, *k*, which takes into account the spatial and temporal distribution of the wake^10^:


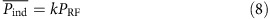


The power required to overcome skin friction and pressure drag, the profile drag *P*_pro_, is given by:


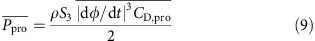


For the mean profile drag coefficient, *C*_D,pro_, we used the published constant value of 1.46 (ref. 39). The inertial power *P*_acc_ required to accelerate the wing in air is^10^:


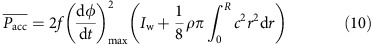


The ratio between Rankin–Froude power and the mechanical power given as the sum of the induced, profile and inertial power is used as the measure of mechanical efficiency *η*:


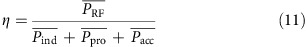


Aerodynamic efficiency is significantly decreased with increasing knockdown, despite the increase in aspect ratio that can enhance the span efficiency, because of the high profile drag compared with induced drag at the low Re (<200). The decrease in lift-generating capacity in stronger phenotypes must lead to modulation of wing kinematics to generate enough aerodynamic force to support their weight and to accelerate; this is a focus of future work.

To compare the aerodynamic torque *T*_a_ during turns with equivalent aerodynamic force *F*_a_ the position of the centre of pressure (that is, the length of moment arm) *x*_m_ is also approximated using the blade element method:


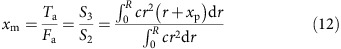


where *x*_p_ is the distance between wing pivot and sagittal plane. Between phenotypes, *x*_m_ does not change, which implies equivalent turning capability with same amount of aerodynamic force generation.

## Additional information

**How to cite this article:** Ray, R. P. *et al.* Enhanced flight performance by genetic manipulation of wing shape in *Drosophila*. *Nat. Commun.* 7:10851 doi: 10.1038/ncomms10851 (2016).

## Supplementary Material

Supplementary Information Supplementary Figures 1-5 and Supplementary Table 1.

## Figures and Tables

**Table 1:** Morphological and performance summary for the control group flies.

**Flight variable**	**Control group mean**±s.d.*	**d*****f***	***F***†	***P*****-value**†
*Morphology*				
Mass (mg)	1.18±0.12	3,78	1.37	0.257
Principal Component 1	—	3,74	**922.56**	**<0.001**
Aspect ratio (single wing mean)	2.52±0.02	3,74	**630.08**	**<0.001**
Relative asymmetry (%)	0.67±0.55	3,71	**5.93**	**0.001**
				
*Flight performance*				
Velocity maximum (m s^−1^)	1.60±0.16	3,78	**14.44**	**<0.001**
Velocity mode (m s^−1^)	0.71±0.33	3,78	0.07	0.977
Tangential acceleration maximum (m s^−2^)	6.60±1.23	3,78	**17.51**	**<0.001**
Tangential acceleration minimum (m s^−2^)	−7.68±2.83	3,78	**12.77**	**<0.001**
Turn radius minimum (mm)	13.2±5.9	3,78	**8.22**	**<0.001**
Turn radius mode (mm)	85.9±28.5	3,78	**6.31**	**<0.001**
Turn rate maximum (deg s^−1^)	1427±378	3,78	**9.03**	**<0.001**
Turn rate mode (deg s^−1^)	112±21	3,78	**4.59**	**0.005**

^*^Flight performance means±s.d. for, CONT flies: *n*=21 individuals. There were 101 flights in the control group in total.

^†^Analysis of variance test for modulation of flight performance across strains of flies with increasing phenotypic severity (*n*=85). Significant results are highlighted in bold and reveal a difference if any one of the experimental groups vary with respect to CONT. For example, when testing for relative asymmetry, only N678 flies differed from CONT. We used the False Discovery Rate method to control the expected proportion of false positives at the 5% level and reduce the likelihood of Type 1 errors^42^,^43^.
