# Supplementary material for: Enhanced flight performance by genetic manipulation of wing shape in Drosophila
Source: Nat Commun. 2016 Mar 1;7:10851. doi: 10.1038/ncomms10851 (PMC4773512; doi:10.1038/ncomms10851)
Supplement: Supplementary Information — Supplementary Figures 1-5 and Supplementary Table 1. [file ncomms10851-s1.pdf]

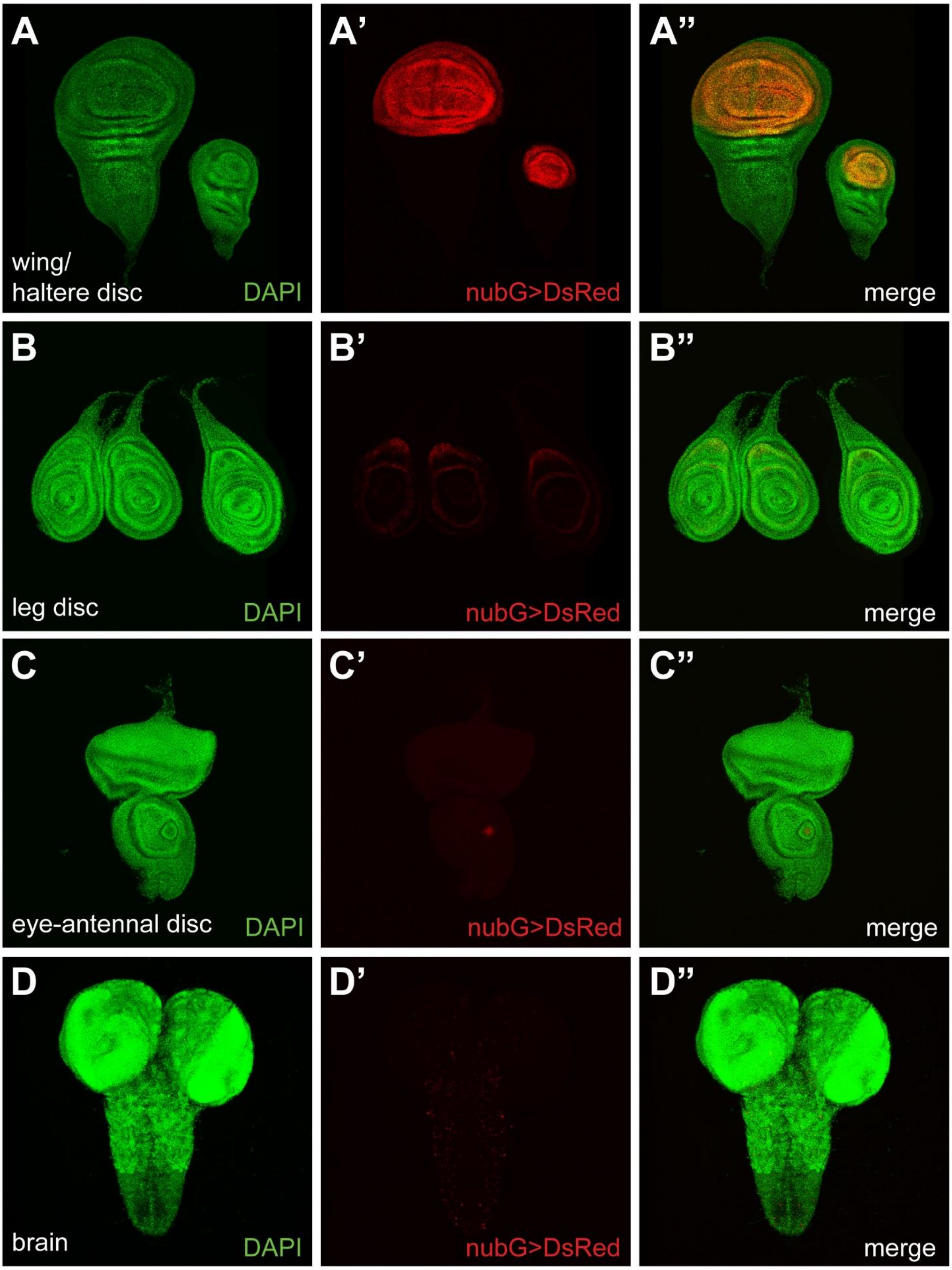
A
A'
A''
wing/
haltere disc
DAPI
nubG>DsRed
merge
B
B'
B''
leg disc
DAPI
nubG>DsRed
merge
C
C'
C''
eye-antennal disc
DAPI
nubG>DsRed
merge
D
D'
D''
brain
DAPI
nubG>DsRed
merge

Supplementary Figure 1.

Expression pattern of *nub-Gal4*. The expression pattern of *nub-Gal4* was assayed by combining it with the reporter UAS-His2ADsRed as in Figure 1. His2ADsRed is localized to the nuclei in the cells where it is expressed (shown in red) and DAPI (shown in green) reveals the overall shape of the tissue. While the gene *nubbin* is expressed in the embryo, this expression is not recapitulated by the Gal4 driver. Expression is only observed in larval and pupal tissues corresponding to places where *nubbin* is normally expressed: the central nervous system and the distal regions of all appendages. Unlike the endogenous gene, the *nub-Gal4* driver is only expressed at high levels in the wing and haltere discs (A) while expression in the legs (B), eye-antennal disc (C) and brain (D) are barely detectable. Consistent with this, mis-expression of potent morphogenetic proteins such as Dpp or the oncoprotein Yorkie under control of *nub-Gal4* only show defects in the wing and haltere, but not in the other appendages (data not shown).

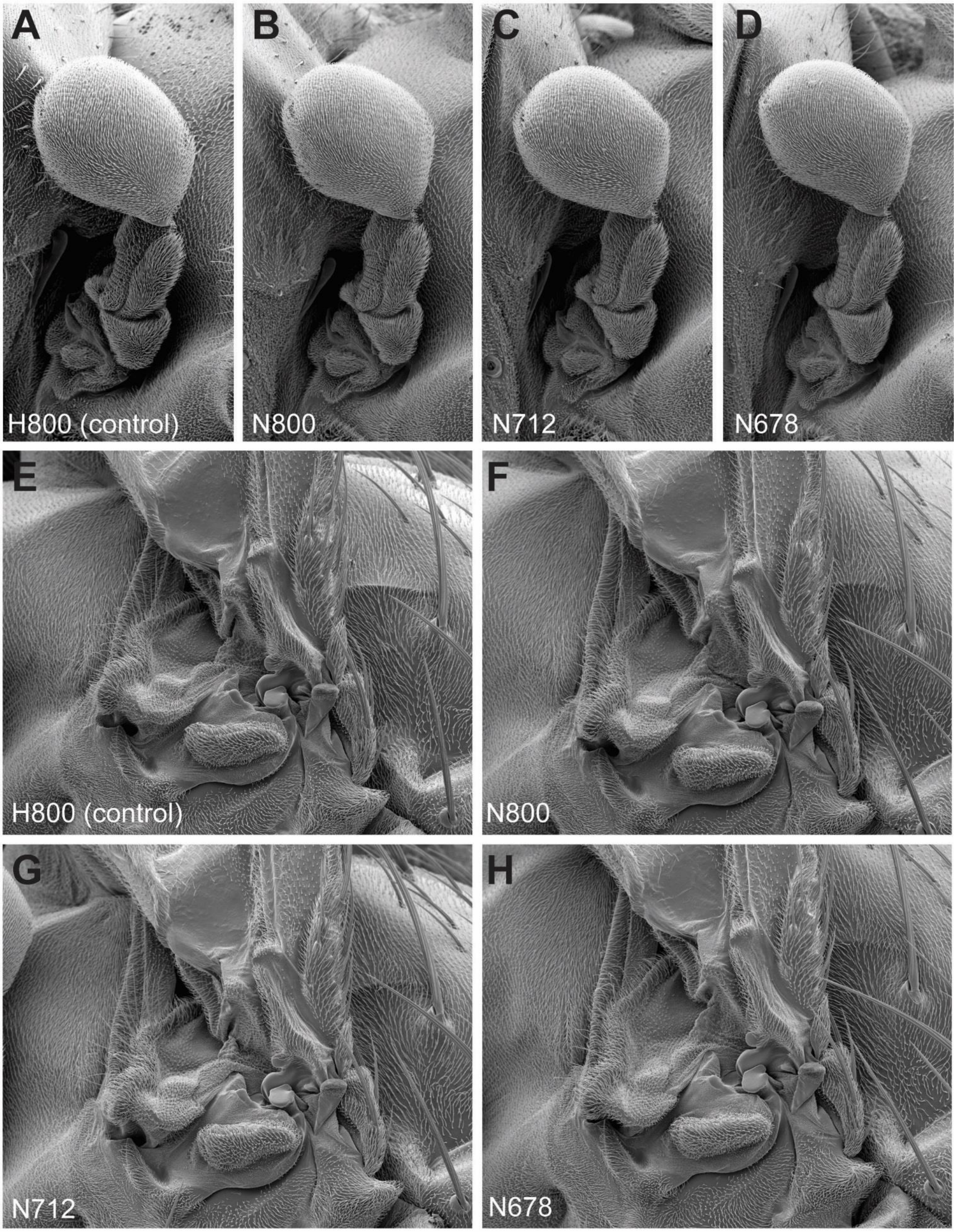
A
H800 (control)
B
N800
C
N712
D
N678
E
H800 (control)
F
N800
G
N712
H
N678

Supplementary Figure 2.

***nub-Gal4>nw-RNAi* does not affect the haltere or wing hinge.** (A-D) Scanning electron micrographs of the adult halteres on flies of the four genotypes used in this study. With *nub-Gal4*, *nw-RNAi* has no effect on hinge morphology regardless of the severity of the associated wing phenotype (cf. Fig. 1C-G). (E-H) Scanning electron micrographs of the ventral surface of the hinge in each of the four genotypes studied. Anterior is to the right, dorsal is up. As with the haltere, *nw-RNAi* has no effect on the morphology of the hinge and associated thorax, consistent with the restricted pattern of *nub-Gal4* expression.

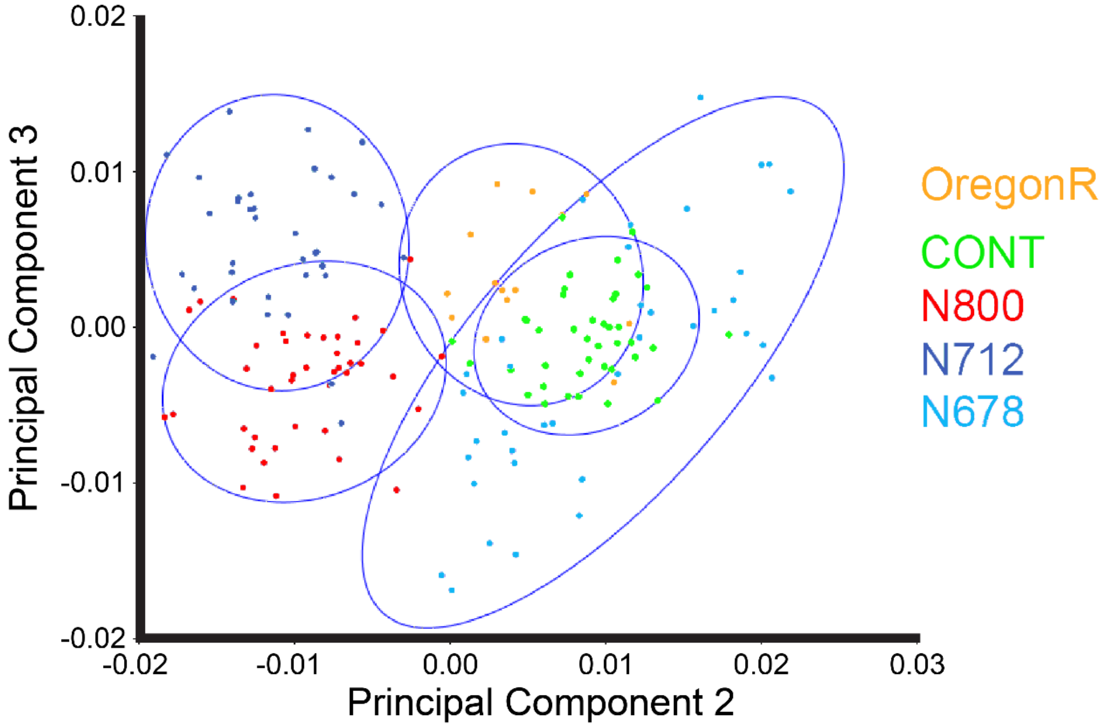

Supplementary Figure 3.

Principal component 2 and principal component 3. PC2 (7.8% variance) and PC3 (2.8% variance) have no monotonic correlation with phenotypic strength and explain only a relatively small proportion of the variation in shape.

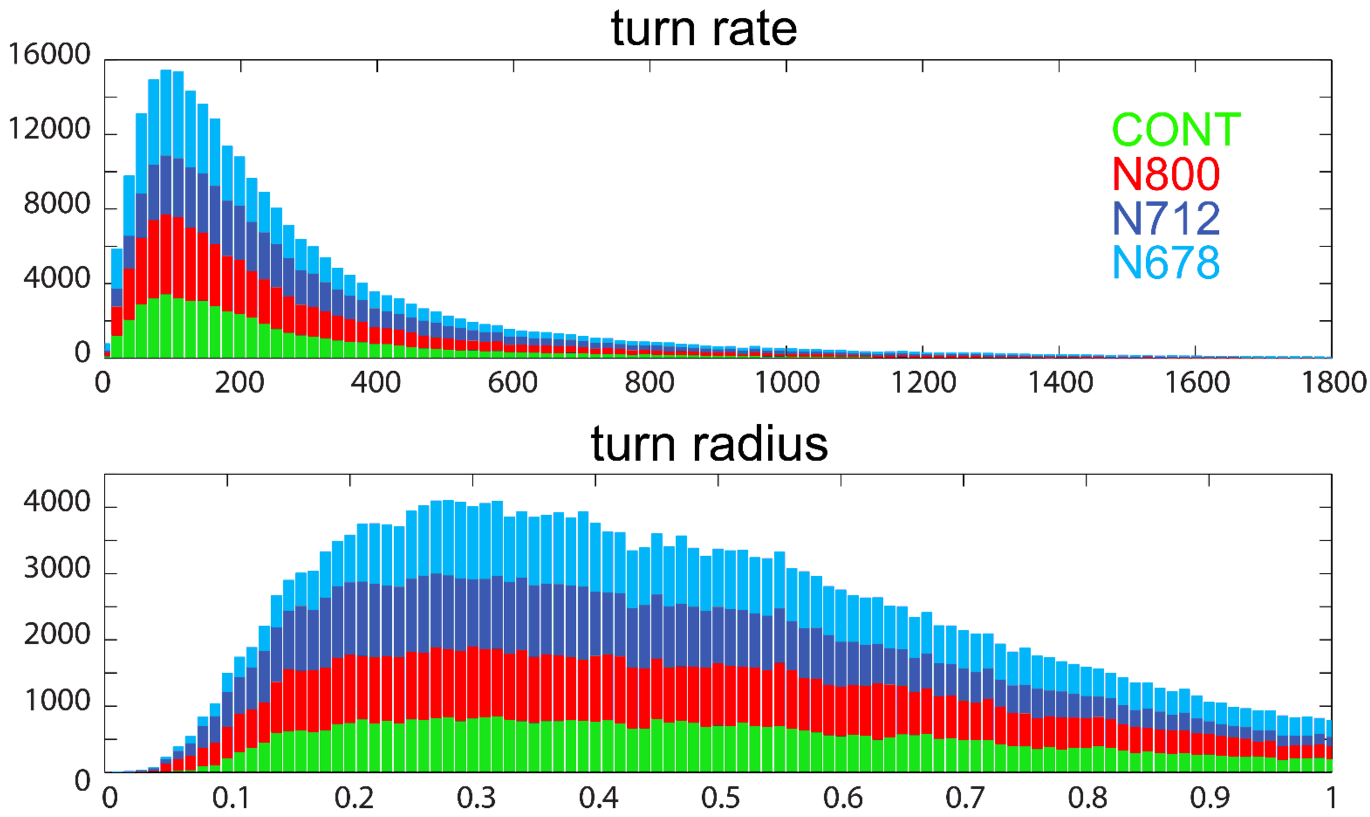

Supplementary Figure 4.

Stacked histograms of turning metrics. Frequency distributions of turn rate (in deg. s^{-1}) and turn radii shorter than one metre (m).

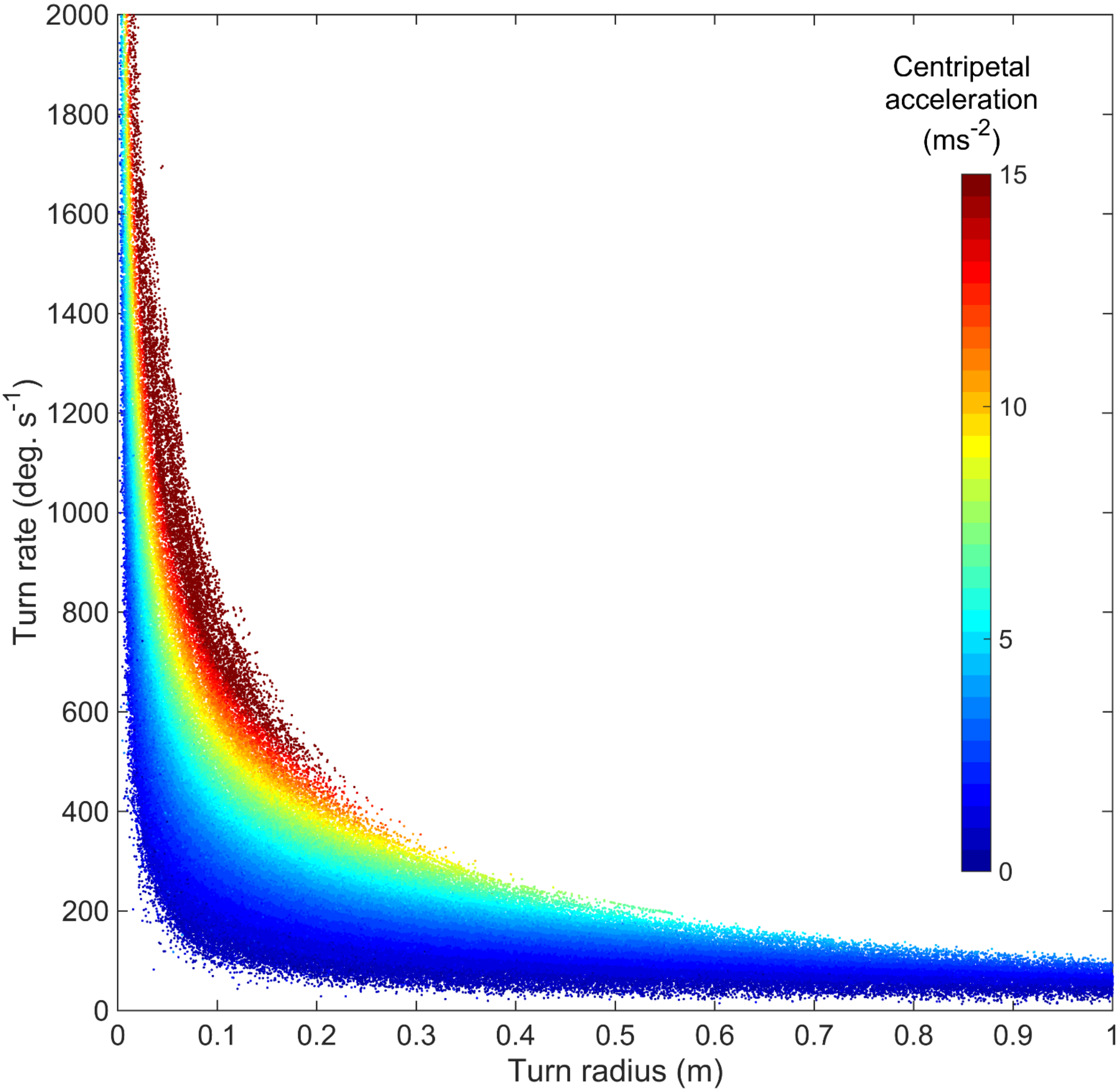

Supplementary Figure 5.

The relationship between turn rate (up to 2000 degrees s^{-1}) and turn radius (up to 1 m) coloured by centripetal acceleration.

Variable	Group	Mean ± S.E.	δ CONT (%)	post hoc ANOVA *p*
**mass (mg)**	CONT	1.18 ± 0.024	-	-
	N800	1.23 ± 0.025	4.8	-
	N712	1.24 ± 0.026	5.6	-
	N678	1.21 ± 0.023	3.0	-
**Principal Component 1 (coefficient)**	CONT	−0.051 ± 0.001	-	-
	N800	−0.015 ± 0.001	−69.8	<0.001
	N712	0.000 ± 0.001	−99.2	<0.001
	N678	0.041 ± 0.001	−179.8	<0.001
**aspect ratio (single wing mean)**	CONT	2.52 ± 0.008	-	-
	N800	2.66 ± 0.008	5.2	<0.001
	N712	2.73 ± 0.009	8.0	<0.001
	N678	3.02 ± 0.008	19.5	<0.001
**wing length (mm)**	CONT	2.12 ± 0.052	-	-
	N800	2.16 ± 0.007	2.0	<0.01
	N712	2.13 ± 0.044	0.7	-
	N678	2.22 ± 0.028	5.1	<0.001
**wing area (single wing mean, mm^2)**	CONT	1.77 ± 0.013	-	-
	N800	1.75 ± 0.014	−1.2	<0.001
	N712	1.66 ± 0.015	−6.6	<0.001
	N678	1.64 ± 0.014	−7.7	<0.001
**wing loading (g m^{-2})**	CONT	332 ± 28	-	-
	N800	352 ± 25	6.0	-
	N712	384 ± 35	15.8	<0.001
	N678	370 ± 35	11.8	<0.001
**second moment of area (non-dimensional, ×10^{-3})**	CONT	286 ± 0.5	-	-
	N800	281 ± 0.5	−1.8	<0.001
	N712	278 ± 0.6	−2.7	<0.001
	N678	273 ± 0.5	−4.7	<0.001
**relative asymmetry (% length)**	CONT	0.64 ± 0.111	-	-
	N800	0.55 ± 0.113	−13.3	-
	N712	0.54 ± 0.127	−14.6	-
	N678	1.15 ± 0.120	81.3	<0.01
**normalised wingbeat frequency (Hz mg$^{1/3}$)**	CONT	315 ± 3.4	-	-
	N800	310 ± 3.4	1.6	-
	N712	314 ± 3.4	−0.2	-
	N678	315 ± 3.4	−0.0	-
**velocity mode (ms^{-1})**	CONT	0.71 ± 0.075	-	-
	N800	0.70 ± 0.077	−1.9	-
	N712	0.67 ± 0.081	−6.2	-
	N678	0.71 ± 0.071	−0.4	-
**velocity maximum (ms^{-1})**	CONT	1.60 ± 0.036	-	-
	N800	1.50 ± 0.037	−6.7	-

	N712	1.52 ± 0.039	−5.4	-
	N678	1.29 ± 0.034	−19.5	<0.001
**tangential acceleration maximum**	CONT	6.60 ± 0.295	-	-
**(ms^{-2})**	N800	6.64 ± 0.303	0.6	-
	N712	8.02 ± 0.319	21.5	<0.01
	N678	4.96 ± 0.282	−24.8	<0.001
**tangential acceleration minimum**	CONT	−7.68 ± 0.549	-	-
**(ms^{-2})**	N800	−8.43 ± 0.563	9.8	-
	N712	−9.75 ± 0.593	27.0	-
	N678	−5.09 ± 0.525	−33.8	<0.01
**centripetal acceleration mode**	CONT	1.68 ± 0.102	-	-
**(ms^{-2})**	N800	1.53 ± 0.105	9.36	-
	N712	1.63 ± 0.111	3.12	-
	N678	1.59 ± 0.098	5.46	-
**centripetal acceleration maximum**	CONT	17.96 ± 0.669	-	-
**(ms^{-2})**	N800	16.89 ± 0.686	5.97	-
	N712	18.77 ± 0.723	−4.49	-
	N678	11.73 ± 0.639	−34.70	<0.001
**turn rate mode**	CONT	112 ± 7.9	-	-
**(deg s^{-1})**	N800	115 ± 8.1	2.4	-
	N712	148 ± 8.5	31.6	<0.01
	N678	110 ± 7.5	−2.1	-
**turn rate max**	CONT	1427 ± 77.2	-	-
**(deg s^{-1})**	N800	1775 ± 79.1	24.3	<0.05
	N712	1729 ± 83.4	21.1	<0.05
	N678	1295 ± 73.8	−9.3	-
**turn radius mode**	CONT	0.086 ± 0.007	-	-
**(m)**	N800	0.071 ± 0.007	−17.2	-
	N712	0.062 ± 0.008	−27.4	-
	N678	0.103 ± 0.007	19.7	-
**√ turn radius min**	CONT	0.112 ± 0.005	-	-
**(m)**	N800	0.089 ± 0.005	−20.8	<0.01
	N712	0.086 ± 0.006	−23.3	<0.001
	N678	0.118 ± 0.005	5.3	-
**wing moment of inertia around wing hinge**	CONT	644 ± 0.60	-	-
**(non-dimensional, $\times 10^{-3}$)**	N800	621 ± 0.41	−3.6	<0.001
	N712	621 ± 0.51	−3.6	<0.001
	N678	599 ± 0.59	−7.0	<0.001
**calculated lift force**	CONT	1.29 ± 0.040	-	-
**(10^{-5} N)**	N800	1.28 ± 0.025	−0.95	-
	N712	1.17 ± 0.024	−9.72	<0.01
	N678	1.16 ± 0.024	−10.41	<0.01
**mechanical efficiency η**	CONT	9.62 ± 0.021	-	-

**(10^{-2})**	N800	9.48 ± 0.015	−1.4	<0.001
	N712	9.38 ± 0.015	−2.5	<0.001
	N678	9.21 ± 0.017	−4.2	<0.001
**length of moment arm (mm)**	CONT	1.95 ± 0.013	-	-
	N800	1.97 ± 0.007	1.1	-
	N712	1.95 ± 0.008	−0.2	-
	N678	1.99 ± 0.009	1.7	-

Supplementary Table 1.

Morphology and performance metric means with pairwise comparisons. Means ± standard error for each group with percentage change from the control group (δ CONT) and the *p*-value from *post hoc* pairwise analysis of variance tests (*n*=85). *Post hoc* tests were made only if the initial analysis of variance tests were significant when including all groups and controlling for multiple tests using Tukey's honestly significant difference criterion. That criterion puts an upper bound (of p = 0.05) on the probability that any comparison will be incorrectly found significant, thereby reducing type 1 errors. Wing area does not include the alula. Wingbeat frequency has been normalised by mass to account for size differences between individuals in the frequency tests.
